# Tumor necrosis factor alpha inhibits *in vitro* bovine embryo development through a prostaglandin mediated mechanism

**DOI:** 10.1186/2049-1891-3-7

**Published:** 2012-03-01

**Authors:** Lauren R Jackson, Char E Farin, Scott Whisnant

**Affiliations:** 1 Department of Animal Science, North Carolina State University, PO Box 7621, Raleigh, NC 27695-7621, USA

**Keywords:** tumor necrosis factor α, bovine, embryo, indomethacin

## Abstract

Mastitis or other infectious diseases have been related to reduced fertility in cattle. Inflammatory cytokines such as tumor necrosis factor α (TNFα) are released in response to infection and may have negative effects on embryo development. In the current study the effect of exposure to TNFα on the development of *in vitro *fertilized bovine embryos was examined. Indomethacin, a prostaglandin synthesis inhibitor, was used to determine if blockade of prostaglandin synthesis would alter the effects of TNFα. Ovaries were obtained from a local abattoir and immature COC were isolated from 2-10 mm follicles, *in vitro* matured and fertilized. After fertilization, groups of presumptive zygotes were randomly placed into either control development medium, medium containing 25 ng/mL TNFα or medium containing 25 ng/mL TNFα plus 1 μg/mL indomethacin. The proportion of blastocysts formed was assessed at day 7 of culture. Fewer embryos exposed to TNFα alone reached the blastocyst stage (17.5 ± 2.4%, P < 0.01) compared with controls (30.5 ± 2.4%) or embryos developed in TNFα plus indomethacin (25.8 ± 2.8%). There was no difference between control embryos and embryos developed in TNFα plus indomethacin. These results indicate that TNFα is inhibitory to the *in vitro* development of bovine embryos and that this inhibition may be mediated by prostaglandins because it can be blocked by indomethacin.

## Background

Mastitis and other inflammatory diseases occur in cattle leading to not only decreased milk production and increased costs but to a reduction in reproductive performance [1,2]. Cytokines, such as tumor necrosis factor α (TNFα), play a role in the immune response and are increased in the serum of cows diagnosed with clinical mastitis [2]. This release of cytokines can have detrimental effects on endometrial and oviductal tissue leading to decreased embryo development. Addition of TNFα to oocyte maturation media *in vitro *reduced the development of bovine cumulus oocyte complexes (COC) to the blastocyst stage [3]. This effect of TNFα could be mediated by prostaglandins. Cyclo-oxygenase-2 (COX2), an inducible enzyme responsible for the first step in prostaglandin production from arachidonic acid is upregulated by cytokines [4]. Exposure of bovine embryos to prostaglandin F2 α (PGF2α) inhibited development to the blastocyst stage *in vitro *[5] and progesterone supplemented cows exposed to PGF2α had reduced embryo quality [6]. Bovine oocytes expressed TNFα mRNA and lower TNFα mRNA levels were associated with more oocytes developing to the blastocyst stage on day 8 [7]. The objective of the current experiment was to determine if addition of indomethacin, an inhibitor of the COX enzyme, could block the detrimental effects of TNFα on development of bovine embryos.

## Materials and methods

### Reagents

Tissue culture medium (TCM-199) was purchased from Gibco-BRL (Grand Island, NY). Fatty-acid free BSA was purchased from Boehringer-Mannheim (Indianapolis, IN). Recombinant bovine TNFα was purchased from Pierce Endogen (Rockford, IL) and reconstituted in sterile saline to a final concentration of 4 ng/mL. All other reagents and media supplements were of tissue culture grade and were obtained from Sigma Chemical Co. (St. Louis, MO). Indomethacin was reconstituted to a 1000X stock solution of 1 μg/mL in sterile absolute ethanol. With the exception of the fertilization medium, all culture media contained 50 μg/mL of gentamicin.

### Maturation, fertilization and development

*In vitro *maturation (IVM), fertilization (IVF) and development (IVD) were performed as the method described by [8]. Ovaries were collected from open cows (based on uterine examination) at a local abattoir and transported to the laboratory in 0.9% saline with 0.75 μg/mL of penicillin (approximately 2 hours). The ovaries were washed in sterile saline upon arrival and cumulus-oocyte-complexes (COCs) were aspirated from 2-10 mm follicles with an 18 gauge needle and syringe. Immature COC were selected and moved to a divided petri dish containing Tyrode medium (TL-Hepes) and washed four times [9]. The COC were placed in maturation medium consisting of M199 with 10% FBS supplemented by 5 μg/mL FSH, 10 μg/mL LH, 1 mg/mL estradiol, 200 μmol/L pyruvate and 50 μg/mL gentamicin for 20 h at 39°C in 5% CO_2_. After maturation, COC were washed and held in fertilization medium containing heparin-supplemented Tyrodes albumin lactate pyruvate (TALP) with 6 mg/mL BSA [8].

Frozen/thawed sperm from the same bull were used for fertilization. Swim-up sperm were collected and used at a concentration of 0.1 × 10^6 ^cell per ml. Gametes were co-incubated for 18 hs 39°C in 5% CO_2. _At 18 h post insemination (hpi) presumptive zygotes were washed six times in TL-Hepes and approximately 25-30 were randomly distributed to one well of a four well culture plate containing the development medium with one of the treatments. All treatments were contained within development medium containing M199, 10% FBS and 50 μg/mL of gentamicin 39°C in 5% CO_2 _and 100% humidity. Development medium was changed at 48 h intervals throughout culture.

Treatments included: Control development medium alone, 25 ng/mL TNFα, 1 μg/mL indomethacin, and 25 ng/mL TNFα plus 1 μg/mL indomethacin. All treatments were replicated four times except indomethacin alone which was replicated twice. Total number of oocytes used in the experiment were control (225), TNFα (232), indomethacin (114) and TNFα plus indomethacin (225).

Development of the embryos was checked at 168 and 216 hpi and evaluated for embryo quality grade and stage of development. Developmental stages were classified as compact morula, early blastocyst, mid-blastocyst, expanded blastocyst and hatched blastocyst. The quality of the embryo was graded as 1 for excellent, 2 for moderate and 3 for poor quality [10].

### Statistical analysis

Data for the percent embryos developing to the blastocyst stage were arc-sin transformed and analyzed by one way ANOVA using PROC GLM of SAS 9.1 (Cary, NC) with Duncan's Multiple Range Test used for mean separation.

## Results

In preliminary experiments, bovine presumptive zygotes were placed into medium containing 0, 10, 25, 50 or 100 ng/mL TNFα and evaluated for development to the blastocyst stage at 168 hpi (2 replicates, n = 60 embryos per treatment). Fewer blastocysts developed in the 25 ng/mL (19.6 ± 2.2%) 50 (18.5 ± 2.7%) and 100 (14.1 ± 1.9%) ng/mL treatments than the 0 ng/mL dose group (27.5 ± 2.6%) so the 25 ng/mL dose was chosen for subsequent experiments as the lowest effective dose. The 1 μg/mL dose of indomethacin was tested in two replicates in the system and had no effect by itself on the percentage of embryos reaching the blastocyst stage (control 25.2 ± 2.1% vs. indomethacin 25.6 ± 2.4%). As ethanol was used for the vehicle for indomethacin, absolute ethanol was tested in two replicates and had no effect on development to the blastocyst stage at 168 hpi at the concentration used (29.7 ± 2.3% control vs. 30.4 ± 2.2% ethanol).

**Figure 1:**
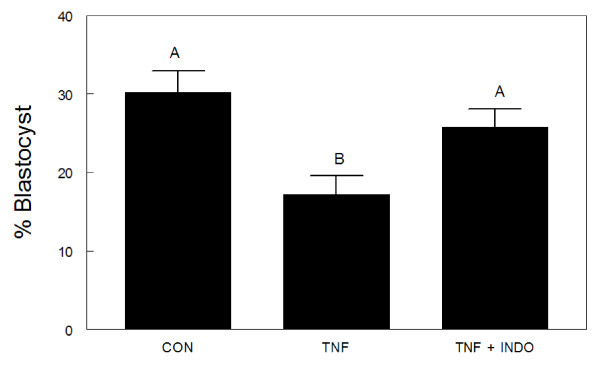
**Comparison of the effects of control medium (CON, 0 ng/mL TNFα, n = 4 replicates, 120 embryos), and addition of 25 ng/mL TNFα (n = 4 replicates, 130 embryos) or 25 ng/mL TNFα plus 1 ug/mL indomethacin (n = 4 replicates, 130 embryos) to medium on the percentage of bovine embryos reaching the blastocyst stage at 168 h post-insemination**.

Treatment with 25 ng/mL TNFα reduced the percentage of bovine embryos reaching the blastocyst stage at 168 hpi (17.2 ± 2.4%) compared to controls (30.2 ± 2.7%) and embryos treated with 25 ng/mL TNFα plus 1 μg/mL indomethacin (25.8 ± 2.3%) (Figure 1). There were no differences in the percentage of embryos in the different classifications among the embryos that reached the blastocyst stage (Table 1).

**Table 1:** Percentage of embryos that reached the blastocyst stage that were classified as early blastocysts (EB), mid blastocyst (MB), expanded blastocyst (ExB), or hatched blastocyst (HB) at 168 h post-insemination.

Treatment	EB	MB	ExB	HB
Control n = 4 replicates, 120 embryos	64.3 ± 3.2%	26.2 ± 2.2%	9.5 ± 0.6%	0
25 ng/mL TNFα n = 4 replicates, 130 embryos	68.8 ± 3.4%	31.2 ± 2.4%	0	0
25 ng/mL TNFα plus 1 μg/mL Indomethacin n = 4 replicates, 130 embryos	40.3 ± 2.5%	43.6 ± 2.5%	16.1 ± 1.4%	0

## Discussion

Soto et al. [3] reported that exposure to TNFα during maturation reduced the percentage of bovine oocytes developing to the blastocyst stage. Exposure of bovine oocytes to TNFα during IVM induced TNFα mRNA expression and reduced the number of oocytes developing to the blastocyst stage on day 8 [7]. Our data are in agreement with these findings. However Soto et al., [3] reported that TNFα added to the incubation media post-fertilization had no effect on the number of oocytes reaching the blastocyst stage on day 8, an observation that is in contrast to our findings. The number of apoptotic cells in blastocysts was increased by incubation with TNFα [3] suggesting that some detrimental effect of TNFα was present. Differences in culture media or conditions may account for contrasting results between the current experiment and that of Soto et al., [3].

The research presented herein indicates that TNFα works through the prostaglandins since the inhibitory effect of TNFα was blocked by co-treatment with indomethacin. In previous work [5], PGF added to the development medium decreased the percentage of bovine embryos reaching the blastocyst stage. On day 7 bovine blastocysts expressed the COX2 gene and TNFα expression was lower in embryos that went on to produce a pregnancy [11] Furthermore, treatment with flunixin meglumine, another prostaglandin synthesis inhibitor, blocked the detrimental effects of oxytocin administration on bovine embryo [12] and addition of a prostaglandin receptor antagonist improved bovine embryo survival [13]. Taken together, these observations support the suggestion that PGs mediate a detrimental effect on blastocyst development. *In vivo*, bovine endometrium and cumulus cells can produce prostaglandins [14,15]. Production of PGF by the bovine oviduct was increased by the presence of TNFα [16] but embryos themselves may also be able to produce PGF in response to TNFα. The results of the current study support the hypothesis that the embryo itself is capable of producing prostaglandins after exposure to TNFα and that this prostaglandin production by the blastocyst is detrimental to its own development, although some prostaglandins may be beneficial to embryo development.

Cows with mastitis had higher concentrations of PGF2α in their milk than cows that did not have mastitis [17] Increased prostaglandin production by blastocysts in response to inflammatory cytokines such as TNFα may explain the decreased fertility that is commonly observed after diseases such as mastitis. Concentrations of TNFα used in the current experiment were higher than those reported in serum of mastitic cows but levels within the uterus and oviduct are unknown

## Competing interests

The authors declare that they have no competing interests.

## Authors' contributions

LJ conducted the oocyte maturation and embryo culture. CF participated in the design of the experiment and provided technical expertise for oocyte maturation and embryo culture. CW conceived the experiment and conducted the statistical analysis. All authors participated in writing the manuscript. All authors have read and approved the final manuscript.
